# Hydroxychloroquine as Prophylaxis for COVID-19: A Review

**DOI:** 10.3389/fphar.2020.605185

**Published:** 2020-12-03

**Authors:** Manuela Monti, Bernadette Vertogen, Carla Masini, Caterina Donati, Claudia Lilli, Chiara Zingaretti, Gerardo Musuraca, Ugo De Giorgi, Claudio Cerchione, Alberto Farolfi, Pietro Cortesi, Pierluigi Viale, Giovanni Martinelli, Oriana Nanni

**Affiliations:** ^1^ Istituto Scientifico Romagnolo per Lo Studio e La Cura Dei Tumori (IRST) IRCCS, Meldola, Italy; ^2^ Dipartimento di Scienze Mediche e Chirugiche, Università di Bologna, Bologna, Italy

**Keywords:** hydroxychloroquine, prophylaxis, COVID-19, review, clinical trials

## Abstract

The impact of the COVID-19 pandemic worldwide has led to a desperate search for effective drugs and vaccines. There are still no approved agents for disease prophylaxis. We thus decided to use a drug repositioning strategy to perform a state-of-the-art review of a promising but controversial drug, hydroxychloroquine (HCQ), in an effort to provide an objective, scientific and methodologically correct overview of its potential prophylactic role. The advantage of using known drugs is that their toxicity profile is well known and there are fewer commercial interests (e.g., expired patents), thus allowing the scientific community to be freer of constraints. The main disadvantage is that the economic resources are almost always insufficient to promote large multinational clinical trials. In the present study, we reviewed the literature and available data on the prophylactic use of HCQ. We also took an in-depth look at all the published clinical data on the drug and examined ongoing clinical trials (CTs) from the most important CT repositories to identify a supporting rationale for HCQ prophylactic use. Our search revealed a substantial amount of preclinical data but a lack of clinical data, highlighting the need to further assess the translational impact of *in vitro* data in a clinical setting. We identified 77 CTs using a multiplicity of HCQ schedules, which clearly indicates that we are still far from reaching a standard of care. The majority of the CTs (92%) are randomized and 53% are being conducted in a phase 3 or 2/3 setting. The comparator is placebo or control in 55 (77%) of the randomized studies. Forty-eight (62%) CTs expect to enroll up to 1,000 subjects and 50 (71%) plan to recruit healthcare workers (HCW). With regard to drug schedules, 45 (58.5%) CTs have planned a loading dose, while 18 (23.4%) have not; the loading dose is 800 mg in 19 trials (42.2%), 400 mg in 19 (42.2%), 600 mg in 4 (8.9%) and 1,200 mg in 1 (2.2%). Forty trials include at least one daily schedule, while 19 have at least one weekly schedule. Forty-one (53.2%) will have a treatment duration of more than 30 days. Awaiting further developments that can only derive from the results of these prospective randomized CTs, the take-home message of our review is that a correct methodological approach is the key to understanding whether prophylactic HCQ can really represent an effective strategy in preventing COVID-19.

## Introduction

Identifying effective antiviral agents to treat and prevent COVID-19 disease is a high priority. The current emergency warrants the urgent development of potential strategies to protect people at high risk of infection, especially cohabitants of diagnosed COVID-19 patients. There are still no drugs approved for disease prophylaxis, despite specific initiatives to accelerate the development of support and evaluation procedures for COVID-19 treatments and vaccines. Thus, the search for effective drugs is mandatory. Treatment is crucial to contrast viral infection, but the prevention of COVID-19 with chemoprophylaxis and vaccination will be the next step in offloading the heavily burdened healthcare system. An efficient approach to drug discovery is to test existing antiviral drugs (Wanget al., 2020). The repositioning of old drugs is an interesting strategy because knowledge of their safety profile, side-effects, posology and drug interactions are well known (Liu et al., 2020). Among several potential candidates, hydroxychloroquine (HCQ) appears to be one of the drugs of choice for large-scale use because of its availability, proven safety and low cost. HCQ is authorized in Europe and the U.S. for different indications. Table 1 provides a summary of the indications, schedules and dosages based on Summary of Product Characteristics (SPC) from the Italian Medicines Agency (AIFA), the European Medicines Agency (EMA), the national EU competent authorities and the Food and Drug Administration (FDA). HCQ is administered as chronic treatment in lupus and rheumatoid arthritis, and is given for a limited period for malaria and photodermatosis. A loading dose of 400–800 mg is frequently used with a daily maintenance dose of 200–400 mg for a period ranging from 3 days until treatment failure, which may means months or years. We thus aimed to review all available resources to gain a state-of-the-art overview of the potential role of HCQ in preventing the spread of COVID-19 and to identify a reasonable schedule for its use as prophylaxis. There are few clinical data on the prophylactic role of HCQ. The rationale to hypothesize the prophylactic use of HCQ derived from first results obtained on around 100 Chinese COVID-19 patients in which the superiority of chloroquine (CQ) over the control group was seen in terms of reduction of exacerbation of pneumonia, duration of symptoms and delay of viral clearance, and absence of severe side-effects (Colson et al., 2020; Gao et al., 2020). This led to China including CQ in its recommendations for the prevention and treatment of COVID-19 pneumonia. The activity of HCQ in viruses is probably similar to that of CQ as the mechanism of action of these two molecules is identical. This, in addition to the better toxicity profile and lower cost of HCQ with respect to CQ, prompted the activation of several CTs on HCQ worldwide. Mainly based on preclinical data, the aim of these trials is to find an effective schedule to prevent COVID-19 infection. Their results will provide the scientific community with reliable and methodologically sound data on which to base future decisions about the use of HCQ in this setting. The need to rapidly find safe and effective treatments for COVID-19 has, in fact, led to questionable data generation and interpretation for the use of HCQ with undesirable downstream effects and a negative impact on public opinion that have caused some competent authorities to discontinue its use in clinical practice. Despite this, numerous studies are now ongoing worldwide using different approaches, different schedules and drug doses. In this paper we review HCQ dosages derived from preclinical and clinical studies and CT repositories.

**TABLE 1:** Authorized indications in Europe and the United States.

Indication	Loading dose	Maintenance dose	Duration
Malaria prophylaxis	800 mg (if not possible to start before exposure)	400 mg/week (1–2 weeks before entering the malaria area)	For 4–8 weeks after leaving the area
Malaria treatment	800 mg followed by 400 mg after 6–8 h	400 mg/day for 2 subsequent days	3 days
—	Single dose of 800 mg (for P. Vivax and P. Falciparum)	—	—
	400 mg/day	200 mg/day	Until failure
—	400–600 mg/day for 6–8 weeks	200–400 mg/day, later 200 mg every other day	Until failure
—	400–600 mg/day	200 mg or 400 mg/day	Until failure
—	—	200 or 400 mg/day	Until failure
—	400–600 mg/day for 2 weeks	200–400 mg/week	Until failure
—	400–600 mg/day for 4–6 weeks	200–400 mg/day	Until failure
—	400–600 mg/dayas loading dose	200–400 mg/day	Until failure
Discoid and systemic lupus erythematosus	400–600 mg/day for 2 weeks	200–400 mg/week	Until failure
—	400–600 mg/day for a few weeks	200–400 mg/day	Until failure
—	400 mg daily until no further improvement is observed	200 mg/day	Until failure
—	—	200 or 400 mg/day	Until failure
—	400–600 mg/day	200 or 400 mg/day	Until failure
—	400–600 mg/day for several weeks if necessary	—	Until failure
—	400–800 mg/day	200–400 mg/day	Until failure
—	400 mg/day	200–400 mg/day	Based on patient’s response
—	400–600 mg/day for 4–6 weeks	200–400 mg/day	Until failure
—	200–400 mg/day; up to 600 mg/day if no response after 1–2 months	Reduction to 100 mg/day for several months, or 200–300 mg/week over several years	Until failure
Photodermatosis	400 mg/day until no further improvement is observed	200 mg/day	—
—	—	400 mg/day	For period of maximum exposure to sunlight
—	—	200 or 400 mg/day	—
—	400–600 mg daily for 4–6 weeks	200–400 mg/day	—
—	—	400–600 mg/day for 7 days before sun exposure	15 days

## Materials and Methods

### Study Design and Objective

The aim of this study was to integrate historical and current preclinical and clinical results and to evaluate all active CTs supporting the prophylactic use of HCQ. For this purpose, we performed a review of the literature and CT databases, including public CT websites.

### Methods

We focused mainly on available preclinical and clinical results on HCQ from PubMed and EMBASE. However, we also searched public CT databases: ClinicalTrials.gov (https://clinicaltrials.gov) (1^st^step), Clinical Trial register (https://www.clinicaltrialsregister.eu) (2^nd^step) and International Clinical Trials Registry Platform (ICTRP) (https://www.who.int/ictrp/en) (3^rd^step). The search terms for all databases were HCQ, COVID-19, prophylaxis, prevention. The search terms for PubMed and EMBASE, selected in various combinations, were HCQ, SARS-Cov-2, COVID-19, preclinical, clinical, prophylaxis, and prevention. We included all published preclinical and clinical data, without limitations, that evaluated the prophylactic role of HCQ.

For the scope of the present work we looked for randomized and non randomized interventional CTs on the use of prophylactic HCQ in subjects who tested negative to COVID-19 or asymptomatic subjects who did not known they were COVID-19-positive. Retrospective and prospective observational studies were excluded from the analysis. We included CT data for 2020 (01/01/2020–October 15, 2020).

## Results

### Preclinical Data as a Rationale for Prophylactic Hydroxychloroquine Posology

Ten articles on prophylactic HCQ posology selected from PubMed are included in the review (Wang et al., 2020; Liu et al., 2020; Gao et al., 2020; Shah et al., 2020; Yao et al., 2020; Zhou, et al., 2020; Garcia-Cremades et al., 2020; Al-Kofahi et al., 2020; Funnel et al., 2020; Maisonnasse et al., 2020). Based on *in vitro* data, several dosages have been proposed but very few results are available on their clinical use. Data on HCQ derive mainly from CQ data as they share a similar chemical structure and mechanisms of action. Both drugs have shown *in vitro* or in animal models to induce an antiviral effect by increasing the endosomal pH, which is crucial for virus-cell fusion. They also interfere with the glycosylation of SARS-COV-2 cell receptors. In addition to their antiviral action, HCQ and CQ have an immunomodulating activity that may synergistically enhance their antiviral effect *in vivo*. *In vitro* studies suggest that the effect on cells is observable when the drug is present before and after the viral inoculum. The choice of HCQ over CQ derives from the former’s greater *in vitro* efficacy. According to a recent study, HCQ may also be active against SARS-COV-2 at lower concentrations than CQ (Liu et al., 2020; Yao et al., 2020). HCQ exhibits a superior *in vitro* antiviral effect compared with CQ and so may be a promising drug for the prevention and treatmentof SARS-CoV-19. According to Liu et al. (2020), both drugs have a similar tissue distribution pattern, with concentrations in the liver, spleen, kidney and lung reaching levels 200- to 700-fold higher than those in plasma. The safe dosage of HCQ is 6–6.5 mg/kg per day, which leads to an HCQ concentration in the above tissues that is like to be able to inhibit SARS-CoV-2 infection. Physiological pharmacokinetic models and *in vitro* data have demonstrated that high concentrations of HCQ can be reached in lung fluid (Yao et al., 2020). HCQ is also an anti-inflammatory agent and can significantly decrease the production of cytokines, responsible for severe COVID-19 inflammatory reactions. According to Yao et al. (2020), HCQ exhibits a superior *in vitro* antiviral and prophylactic activity compared to CQ when it is added prior to the viral challenge. The authors report that HCQ-calculated lung, blood and plasma concentrations rapidly increase and reach steady state following the initial loading dose and subsequent maintenance doses.

Al-Kofahi et al. (2020) simulated potential HCQ dosing regimens for pre-exposure and post-exposure prophylaxis, their data suggesting that higher HCQ doses than those recommended for chemoprophylaxis of malaria may be required. Recent studies on in hamster and non human primates showed no protection against infection derived from pre-exposure prophylactic HCQ treatment (Funnel et al., 2020; Maisonnasse et al., 2020).

### Clinical Results

At the time of writing the present review (October 15, 2020), there were some preliminary data on the use of prophylactic HCQ in randomized CTs; Boulware et al. (2020) reported their results on HCQ as a post-exposure prophylaxis for COVID-19 vs. placebo. The study enrolled 821 asymptomatic individuals exposed to confirmed Covid-19 subjects who were randomly assigned to receive placebo or HCQ. The incidence of a new illness compatible with COVID-19 did not differ significantly between the two arms. Side-effects were more common with HCQ than with placebo, but no serious adverse reactions were reported. Rajasingham et al. reported the results of a pre-exposure prophylaxis with HCQ in a randomized study of healthcare workers (HCW) in which 1,483 subjects were randomized to receive HCQ (HCQ 400 mg weekly *vs*. HCQ 400 mg twice weekly) vs. placebo. The authors did not find a significant reduction in COVID incidence in any of the three arms. Abella et al. carried out a randomized double-blind placebo-controlled trial on a HCW population, reporting no significant differences in infection rates between participants taking HCQ or placebo. The trial was stopped early for futility before reaching the planned enrollment. With regard to safety issues, Abella et al. did not report any grade 3 or four adverse events or cardiac events. Rajasingham et al. reported one cardiac event that was potentially HCQ-related.

Given the lack of clinical data on HCQ, we moved our search to CTs to identify the prophylactic scheme chosen in important clinical centers around the world to contrast COVID-19 disease. We therefore selected interventional prospective CTs on prophylactic HCQ in subjects not documented to have COVID-19 or to be SARS-CoV-2-positive. Trials enrolling positive subjects, even when a symptomatic, were excluded because in such cases HCQ is considered treatment and not prophylaxis. We considered both pre-exposure and post-exposure settings. Search results (Table 2) from www.clinicaltrials.gov highlighted 68 ongoing CTs activated from January 01, 2020 to October 15, 2020, 51of which met study requirements. Seventeen CTs were excluded because they were observational (6 CTs) or included COVID-19 patients (11 CTs). We then performed the same search in other databases, excluding CTs already captured in the previous search and excluding those that failed to meet study requirements. We identified 20 CT sin the EU Clinical Trials Register (European database), 12 of which were considered for the present work. As 3rd step, we searched for CTs on the World Health Organization International Clinical Trials Registry Platform (WHO ICTRP), which is automatically updated by the American and European databases each week, but also interfaces with other databases worldwide every 4 weeks. We identified 79 CTs, 14 of which are included in our analysis. A total of 77 CTs were identified (Supplementary Table S1). Table 3 describes CT design: 92% of all studies are randomised and 53% are phase 3 or 2/3. The comparator is placebo or control in 55 (77%) of the randomised studies. Among the remaining 16 CTs, 3 studies are comparing different schedules of HCQ, five are evaluating HCQ in association with vitamins and/or zinc or compared HCQ with vitamins and/or zinc, 3 administer HCQ in combination with lopinavir/ritonavir, 2 HCQ with azythromicyn, 1 HCQ with bromhexine, 1 HCQ with emtricitabine/tenofovir disoproxil and 1 HCQ*vs*.ivermectin vs. zinc vs. povidoneiIodine vs. vitamin C. As reported in Table 4, 48 (62%) CTs expect to enroll up to 1,000 subjects, 50 (65%) plan to enroll HCW, and only 13 (17%) plan to recruit contacts (no HCW). With regard to drug schedule, 45 (58.4%) CTs are using a loading dose while 18 CTs (23.3%) are not. This information is unavailable for 15 (19.5%) CTs.The loading dose is 800 mg in 19 (42.2%) trials, 400 mg in 19 (42.2%), 600 mg in 4 (8.9%) and 1,200 mg in 1 (2.2%). Forty CTs include at least one daily schedule of HCQ and 19 at least one weekly schedule. Forty-one (53.2%) trials have a treatment duration of more than 30 days. Given the substantial schedule variability, the most indicative value may be dose intensity (total dosage divided by the total administration period), which ranges from a daily dose of 37 mg up to 800 mg (Supplementary Table S1).

**TABLE 2:** CT database search.

Step	Database	No.clinical trials	CTs^*^	Search specifications
1	Clinical trials.gov	68	51	From 68 to 51
2	EU Clinical trial register	20	12	From 20 to 12 (no trials captured with step 1 or without study requirements)
3	ICTRP	79	14	From 79 to 14 (no trials captured with steps 1 and 2 or without study requirements)

* None of the trials were selected more than once.

**TABLE 3:** Clinical trial design.

CT phase	No. CTs (%)	No. randomized CTs (total no. 71) (92%)	Standard arm placebo	Standard arm control	Standard arm other drug
Phase 1	1 (1.3)	1 (1.3)	0	0	1 (1.3)
Phase 2	12 (15.6)	9 (11.7)	2 (2.6)	5 (6.5)	2 (2.6)
Phase 3–2/3	53 (68.8)	53 (68.8)	26 (33.8)	15 (19.5)	12 (15.6)
Phase 4	4 (5.2)	4 (5.2)	4 (5.2)	0	0
NA/UN	7 (9.1)	4 (5.2)	1 (1.3)	2 (2.6)	1 (1.3)

CT, clinical trial; NA, not applicable; UN, unknown.

**TABLE 4:** Clinical trial characteristics.

No. subjects (range)	No. CTs (%)
≤200 (60–200)	10 (13.0)
201–500 (206–500)	25 (32.5)
501–1,000 (530–1,000)	13 (16.9)
1,001–2,000 (1,100–2000)	14 (18.2)
2,001–10,000 (2,250–6,400)	11 (14.3)
>10,000 (10,990–40,000)	3 (3.9)
CT population	No. CTs (%)
HCW	50 (64.9)
Contacts	13 (16.9)
Vulnerable patients	8 (10.4)
Other	6 (7.8)
Maintenance dose	No. CTs (%)
200 mg/day	18 (23.4)
400 mg/day	18 (23.4)
600 mg/day	3 (3.9)
400 mg/week	18 (23.4)
Other	21 (27.3)
Treatment duration (range)	No. CTs (%)
Max 5 days (4–5)	9 (11.7)
Max14 days (10–14)	6 (77.9)
Max 21 days (21)	2 (2.6)
Max 30 days (28–30)	5 (6.5)
Max 60 days (40–60)	21 (27.3)
>60 days (77–180)	20 (26.0)
Other/not reported (/)	14 (18.2)

CT, clinical trial; HCW, healthcare worker.

## Discussion

The role of HCQ in the prevention of SARS-CoV-2 and its optimal prophylactic dosage have yet to be clarified. Although *in vitro* data suggest that HCQ may be effective in preventing infection thanks to its mechanism of action, robust clinical evidence is still missing. On the basis of preclinical results, HCQ can be given at a maximum dose of 1,200 mg daily (Shah et al., 2020). Garcia-Cremades et al., (2020) performed pharmacokinetic simulations, observing that 800 mg/day either loaded upfront or as 400 mg b. i.d., appeared to have good efficacy on viral load. A single dose of HCQ 800 mg reached a lung tissue concentration more than 20-fold higher than EC50 (half maximal effective concentration) values needed to inhibit SARS-CoV-2 in the lung on day 1 (Yao et al., 2020). Given that the half-life of HCQ in blood after a single dose of 200 mg is 22 days (hydroxychloroquine sulfate tablets, 2020), a single dose each week or even every three weeks should be sufficient to prevent SARS-CoV-2-induced lung damage. The first results arrived from China, but the schedule for their prophylactic use was empirical and heterogeneous, causing further dilemma among western healthcare professionals. Results from interventional CTs on the prophylactic use of HCQ are very limited. Our search of PubMed and EMBASE revealed three randomized trials, one investigating post-exposure prophylaxis in individuals exposed to confirmed Covid-19 subjects (Boulware et al., 2020) and two investigating pre-exposure prophylaxis in healthcare workers (Abella et al., 2020; Rajasingham et al., 2020). Cohen et al. pointed out several limitations of Boulware’s trial such as the specificity of participant-reported symptoms, no monitoring of adherence to the intervention and, in particular, the long delay between perceived exposure to SARS-CoV-2 and the start of HCQ (Cohen, 2020). Gurjar et al. hypothesized that the results of Boulware’s study may simply have been affected by the timing of therapy with respect to exposure (Gurjar and Agarwal, 2020). Furthermore, the trials by Rajasingham and Abella may have been limited by their small sample size and insufficient statistical power.

A search of international CTs databases has shown that numerous large randomized clinical trials promoted by prestigious institutions are now active. It is hoped that their findings will provide clear and comprehensive information on the efficacy of HCQ in preventing SARS-CoV 2 infection. However, enrolling participants in trials on HCQ became a challenge in May 2020 when EMA (EMA, 2020), FDA and local regulatory authorities issued warnings on HCQ safety derived from some retrospective studies.

During this exceptional period, drug repositioning is one of the strategies that has been adopted in the fight against SARS-COV-2. Following the concept of drug repositioning, Colson et al. (2020), Gao et al. (2020) and Zhou et al. (2020) published results supporting the prophylactic use of HCQ because of its *in vitro* ability to interfere with cellular receptors for COVID-19 and block virus fusion with host cells. The advantage of using existing drugs is that their toxicity profile is well known and there are fewer commercial interests (e.g.,expired patents), thus freeing the scientific community from legal and financial constraints. The downside is that economic resources are often insufficient to promote large multinational CTs. The fact that 98% of the CTs identified in the present review are no profit bears witness to this. CTs are needed to assess the translational impact of *in vitro* data in a clinical setting as preclinical findings do not always translate to real efficacy *in vivo.* Other important concerns are toxicity and drug interaction. Although HCQ is a relatively safe drug, some caution is needed for its use because of QT prolongation. It is therefore important to promote randomized CTs on HCQ with control or place bo as there is still no standard of care. We must, however, take into account that large randomized CTs are not always feasible or ethical, and that patients may need to be treated empirically during times of uncertainty. 95% of the interventional CTs we identified are randomized, highlighting the need for methodologically sound studies. In the present study, we focused our attention on CTs evaluating the prophylactic use of HCQ, excluding studies with COVID-19-positive subjects because HCQ would then have been considered a treatment. We selected CTs including subjects who were confirmed negative or asymptomatic individuals who had not been tested for the virus. Of note, positivity to COVID-19 cannot be totally excluded as tests have limits, and there is a high percentage of positive a symptomatic subjects whose prognosis is nevertheless good. Another limit of the present study is that we did not differentiate between “pre-exposure prophylaxis” and “post-exposure prophylaxis”. However, we are aware of the fact that, in addition to dosage, the timing of prophylactic therapy is probably one of the main issues affecting the ability of HCQ to contain the risk of being infected or of developing the disease.

From the point of view of HCQ safety, it must be remembered not to exceed dosages administered for other indications and to adopt existing authorized schedules whose toxicity profile and drug interactions are known. Although non-severe safety concerns were reported in the three randomized studies by Boulware, Rajasingham and Abella, a significant increase in common and mild HCQ-related adverse events were observed. With regard to treatment schedules, the preclinical data of Colson et al. revealed that it may be necessary to administer a loading dose followed by a maintenance dose (Colson et al., 2020). A loading dose of 400–800 mg was used in 84% of the CTs we considered in the present study. A loading dose is often mandatory in the majority of the authorized indications for the use of HCQ (Table 1). Maintenance schedules are heterogeneous, ranging from 200 to 600 mg/day (50.6%) and from 200 to 400 mg/week (24.7.%). Such variability is a clear indication of uncertainty. Treatment duration is even more heterogeneous and cannot be justified with available *in vitro* data. This multiplicity of schedules indicates that we are still a long way from reaching a standard of care and highlights the risk of conflicting results from CTs (Bienvenu et al., 2020). During the COVID-19 emergency, HCQ has been prescribed as off-label treatment, with several differences between countries. The question of HCQ for COVID-19 prevention has got somewhat out of hand due to interference from politicians and nonscientists via social media and non scientific journals. However, as Kim et al. stated, “it is our responsibility as clinicians, researchers, and patient partners to promote proper and rigorous interpretation of results, particularly in our interactions with the non-scientific community. We must consider the societal implications of published work in these unprecedented times” (Kim et al., 2020). The role of prophylactic HCQ in SARS-CoV-2 and the definition of the optimal dosage are two important issues requiring immediate attention. In the absence of robust data, it seems premature to recommend HCQ as a prophylactic panacea for COVID-19. Results from the ongoing randomized CTs are thus eagerly awaited to see whether HCQ can really prove effective against the virus. Certainly, in this period of global emergency, regulatory agencies have also defined guidelines based on empirical and not methodologically flawless data. Aside from the risk of misinterpretation, the unrestrained and uncontrolled use of HCQ has also resulted in a shortage for patients with authorized indications such as lupus, rheumatoid arthritis or malaria. Several other agents are currently under investigation as pre- or post-exposure prophylaxis for COVID-19, among which ivermectin, emtricitabine plus tenofovir alafenamide or tenofovir disoproxil fumarate, lopinavir/ritonavir, and nitazoxanide. Supplements such as zinc, vitamin super B-complex, vitamin C, and vitamin D are also being evaluated, and approaches using monoclonal antibodies targeting SARS-CoV-2 and convalescent plasma are being developed. However, there are still no recommendations from NIH’s COVID-19 Treatment Guidelines Panel of Experts for the use of any of the above agents except in a clinical trial setting (www.covid19treatmentguidelines.nih.gov).

In conclusion, in expectation of further developments that can only derive from large prospective randomized CTs, the take-home message of our research is that a correct methodological approach is the key to understanding whether prophylactic HCQ can really represent an effective strategy in preventing COVID-19. Thus, a meta-analysis of the results of the ongoing randomized CTs would serve to lend weight to their results and make a large-scale use of prophylactic HCQ justified.

## Author Contributions

All authors made an intellectual and direct contribution to the drafting of this article, and all read and approved the present version of the manuscript for publication.

## Conflict of Interest

The authors declare that the research was conducted in the absence of any commercial or financial relationships that could be construed as a potential conflict of interest.

## Abbreviations

Abbreviations: CQ, chloroquine; CTs, clinical trials; HCQ, hydroxychloroquine; HCW, healthcare workers; SPC, summary of product characteristics.
